# Supplementary material for: Tumor Microenvironment-Responsive Magnetic Nanofluid for Enhanced Tumor MRI and Tumor multi-treatments
Source: Pharmaceuticals (Basel). 2023 Jan 23;16(2):166. doi: 10.3390/ph16020166 (PMC9961229; doi:10.3390/ph16020166)
Supplement: Supplementary file 1 [file pharmaceuticals-16-00166-s001.zip › pharmaceuticals-2102596-supplementary.pdf]

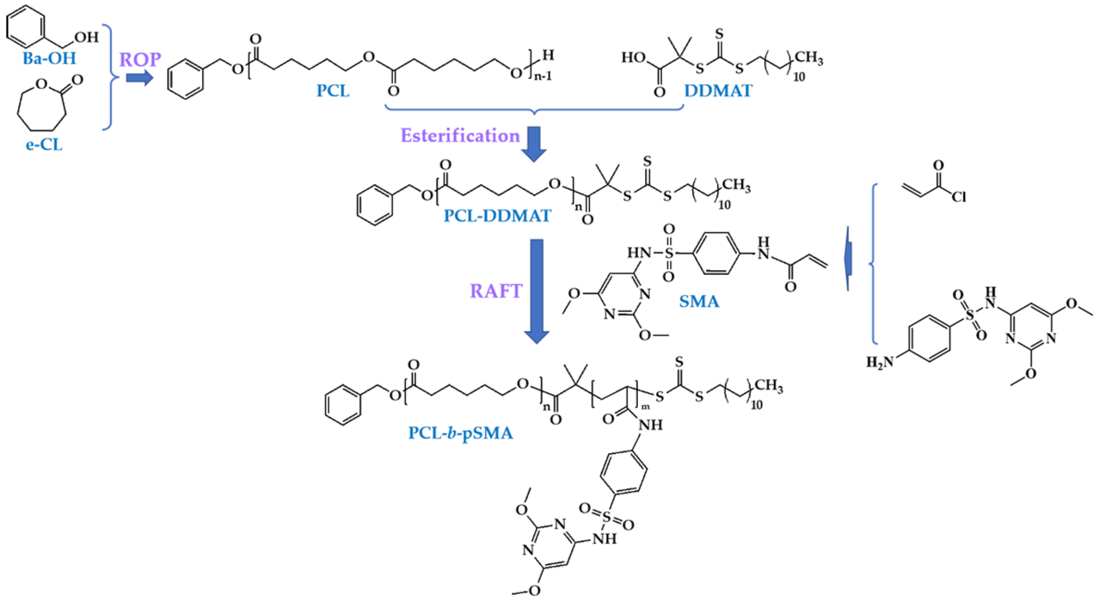

Figure S1: Synthetic scheme of PCL-*b*-pSMA

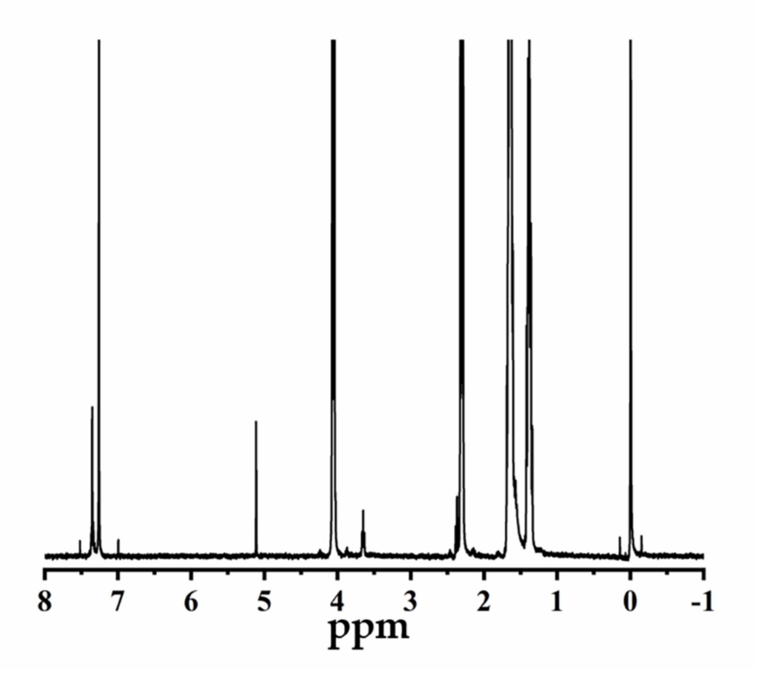

Figure S2: ^{1}H NMR spectrum of PCL

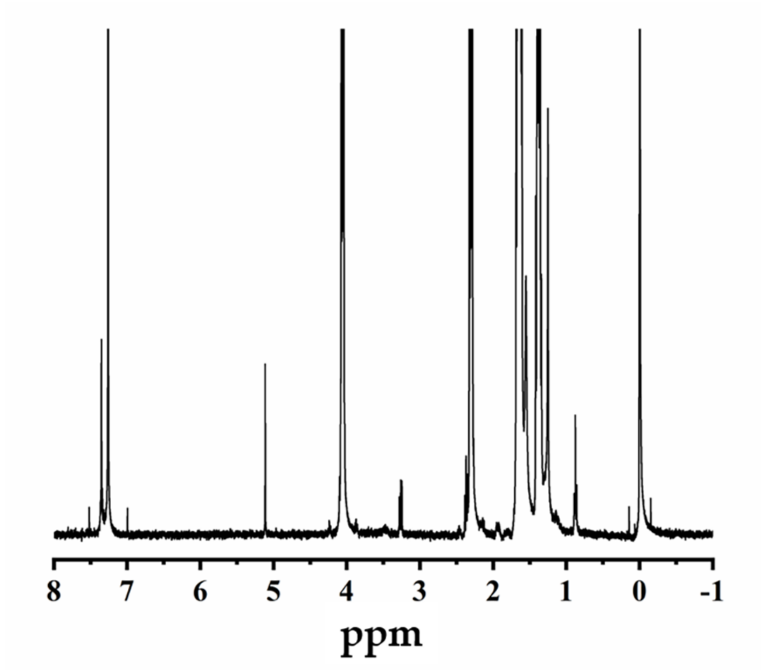

Figure S3: ^{1}H NMR spectrum of PCL-DDMAT

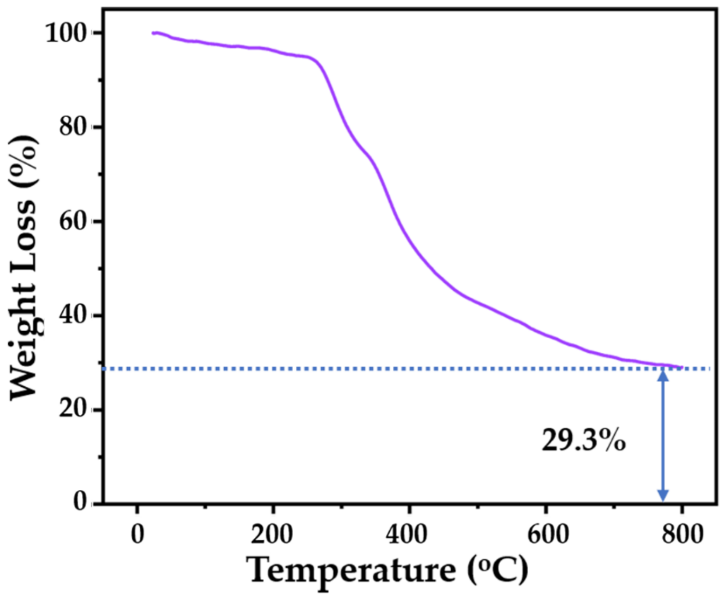

Figure S4: The content of Fe_3O_4 in tumor microenvironment-responsive MNFs

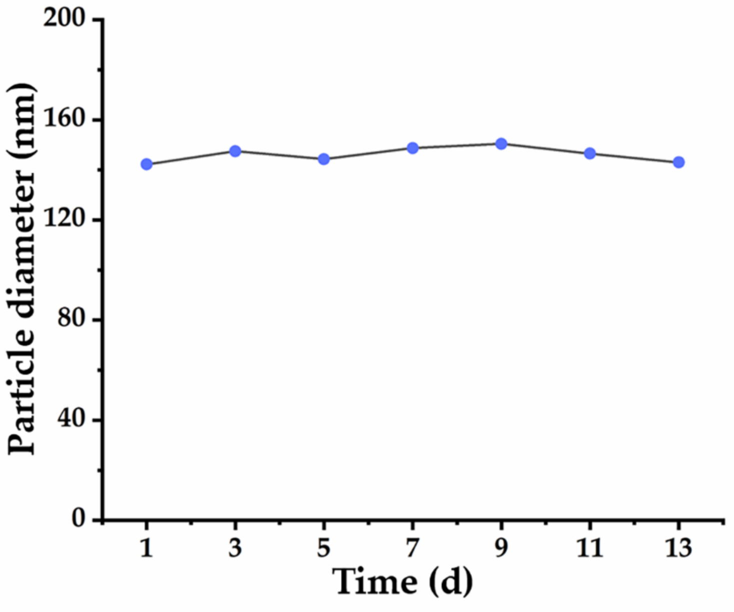

Figure S5: The time-dependent hydrodynamic diameter of tumor microenvironment-responsive MNFs in physiological condition

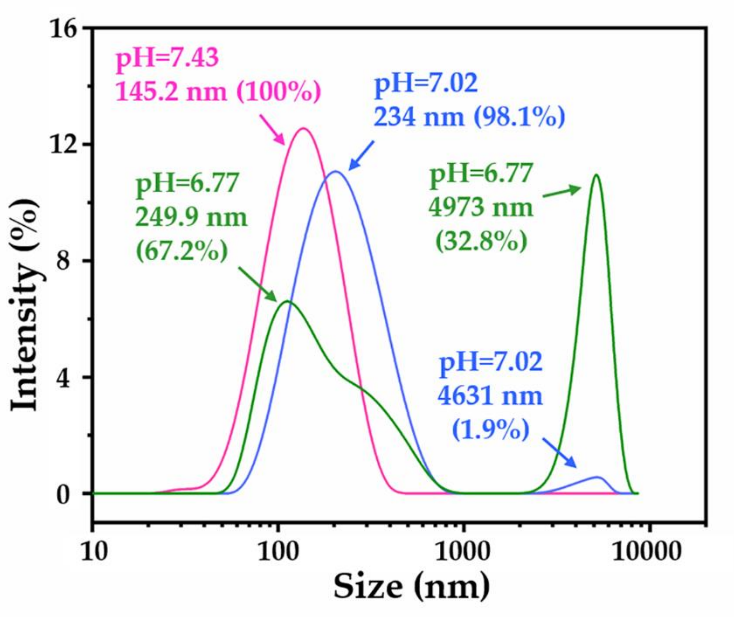

Figure S6: Particle sizes of tumor microenvironment-responsive MNFs in different pH values

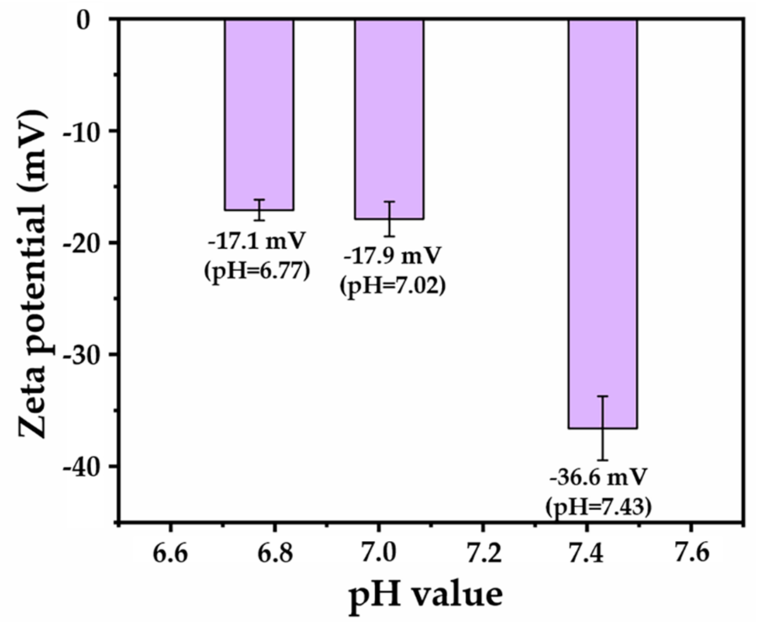

Figure S7: The content of Fe_3O_4 in tumor microenvironment-responsive MNFs

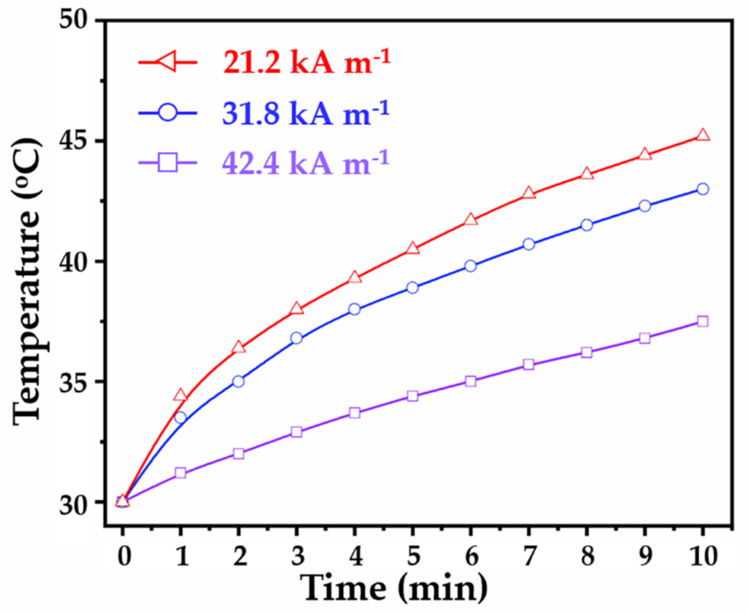

Figure S8: Time-dependent temperature curves of tumor microenvironment-responsive MNFs under AMF with different $H_{applied}$
